# Fully digital workflow, integrating dental scan, smile design and CAD-CAM: case report

**DOI:** 10.1186/s12903-018-0597-0

**Published:** 2018-08-07

**Authors:** Miguel Stanley, Ana Gomes Paz, Inês Miguel, Christian Coachman

**Affiliations:** 1 Private Practice at White Clinic, Rua Dr. António Loureiro Borges. Edif. 5, 1° Andar Arquiparque; 1495-131 Algés-, Lisbon, Portugal; 2 Private Practice, Rua Bento de Andrade, São Paulo, SP 116 Brazil; 3 Endodontic Department at FMDUL, Private Practice at White Clinic, Rua Dr. António Loureiro Borges. Edif. 5, 1° Andar Arquiparque, 1495-131 Algés, Portugal

**Keywords:** Digital workflow, Digital planning, Digital smile design, Intraoral scanner, 3D printer, CAD-CAM, Minimal preparation, Monolithic lithium disilicate ceramic

## Abstract

**Background:**

This report is a presentation of a clinical case that follows a full digital workflow.

**Case presentation:**

A 47-year old man presented with pain in the TMJ (temporomandibular joint) and whose aesthetic concern was having a chipped maxillary central incisor veneer. The concern was solved following a fully digital workflow: it was applied the digital smile design protocol, as well as CAD-CAM monolithic lithium disilicate ceramic veneers and crowns (following a minimal invasive preparation approach). The aim of this rehabilitation was to solve a loss of vertical dimension, subsequent aesthetics and temporomandibular joint disorders.

**Conclusion:**

Thanks to the evolution of technology in dentistry, it is possible to do a full digital case and solve problems such as loss of vertical dimension successfully. Nevertheless, more clinical studies are needed to obtain consistent results about the digital work flow compared to the conventional technique in loss of vertical dimension cases.

## Background

Digital work flow in dentistry has increased in recent years due to the headway made in technologies such as intraoral scanners and software programs, which have contributed to improve communication between the clinician and the dental technician. The Digital Smile Design (DSD) is a digital tool which provides, from a facial perspective, rehabilitative aesthetic planning, better communication between specialists and an improvement in the expected outcome of the treatments [1]. A dynamic documentation of the smile is an important step in the 2D/3D digital smile design process that can be performed in a entire digital flow and will help in the rehabilitative procedures. The advantages of using video documentation are that it facilitates and simplifies the documentation process, improves smile design, facial analysis, treatment planning, team communication and patient education [2]. The DSD could be converted into a conventional or virtual diagnostic model to facilitate subsequent clinical treatments, i.e. CAD-CAM restorations [3–7]. The preparations for minimally invasive treatments have become easily achievable in restorative dentistry because of the combination of the adhesive technique with restorative materials featuring translucent properties. Materials like lithium disilicate ceramic [8–11] have properties like those existing in natural teeth so they have presented positive outcomes [12, 13].

Another important tool that integrates the digital workflow are the intraoral scanners. These are powerful devices that allow an immediate determination of the quality of the impression and have the capacity to easily send the models to the laboratory using e-mail, thus reducing expense and time [14]. Nevertheless, there is limited literature about intraoral scanner potential capturing high quality impressions [15–24].

Computer-aided design (CAD) software is an essential tool since it is responsible for guiding robotic devices which create objects and assemblies in a virtual environment [25].

This report is a presentation of a clinical case that follows a full digital workflow. After a minimal invasive preparation approach, the digital smile protocol, CAD-CAM monolithic lithium disilicate ceramic veneers and crowns was used to solve a loss of vertical dimension, subsequent aesthetics and temporomandibular joint disorders.

## Case presentation

**Fig. 1:**
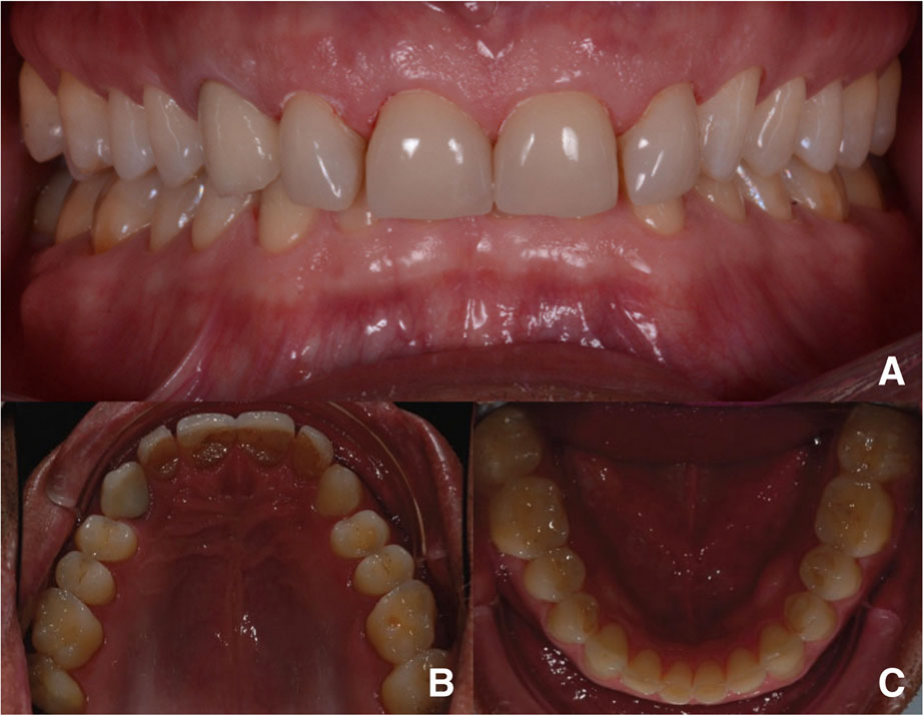
**a** Pretreatment intraoral photo: Front view. **b** Pretreatment intraoral photo: Occlusal view upper jaw. **c** Pretreatment intraoral photo: Occlusal view lower jaw

**Fig. 2:**
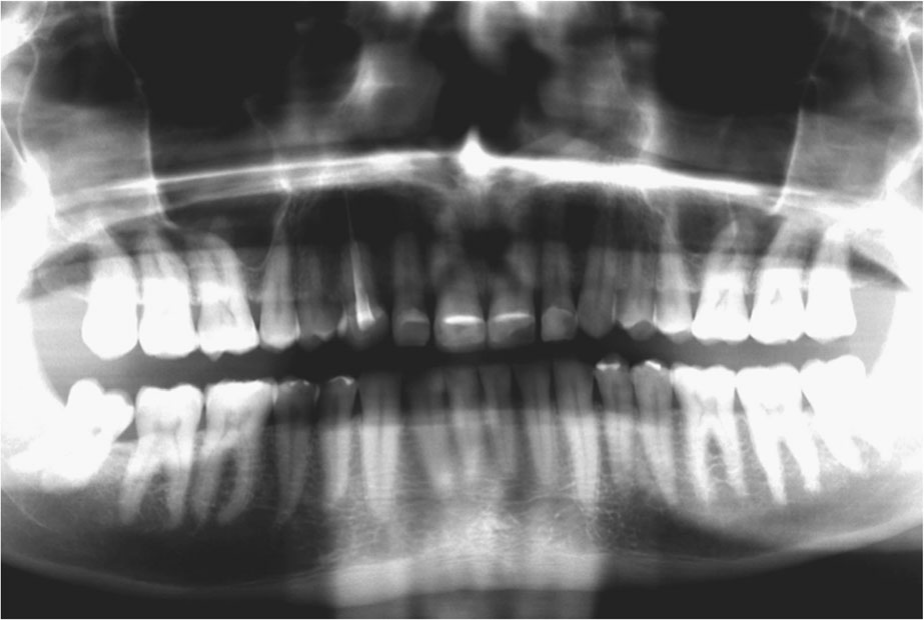
Initial Panoramic (2015)

In 2015, a 47-year old man presented with pain in the TMJ (temporomandibular joint) and whose aesthetic concern was having a chipped maxillary central incisor veneer, as seen in Fig. 1a, b and c. After a clinical and radiographic analysis, as seen in Fig. 2, a loss of the vertical dimension and tooth ware, caused by bruxism, was diagnosed.

**Fig. 3:**
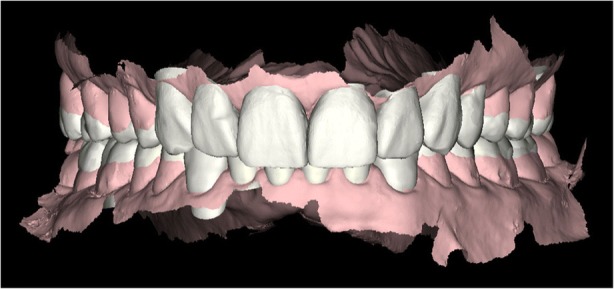
Initial digital intraoral scanner with Carestream® 3500; Rochester, NY, USA

**Fig. 4:**
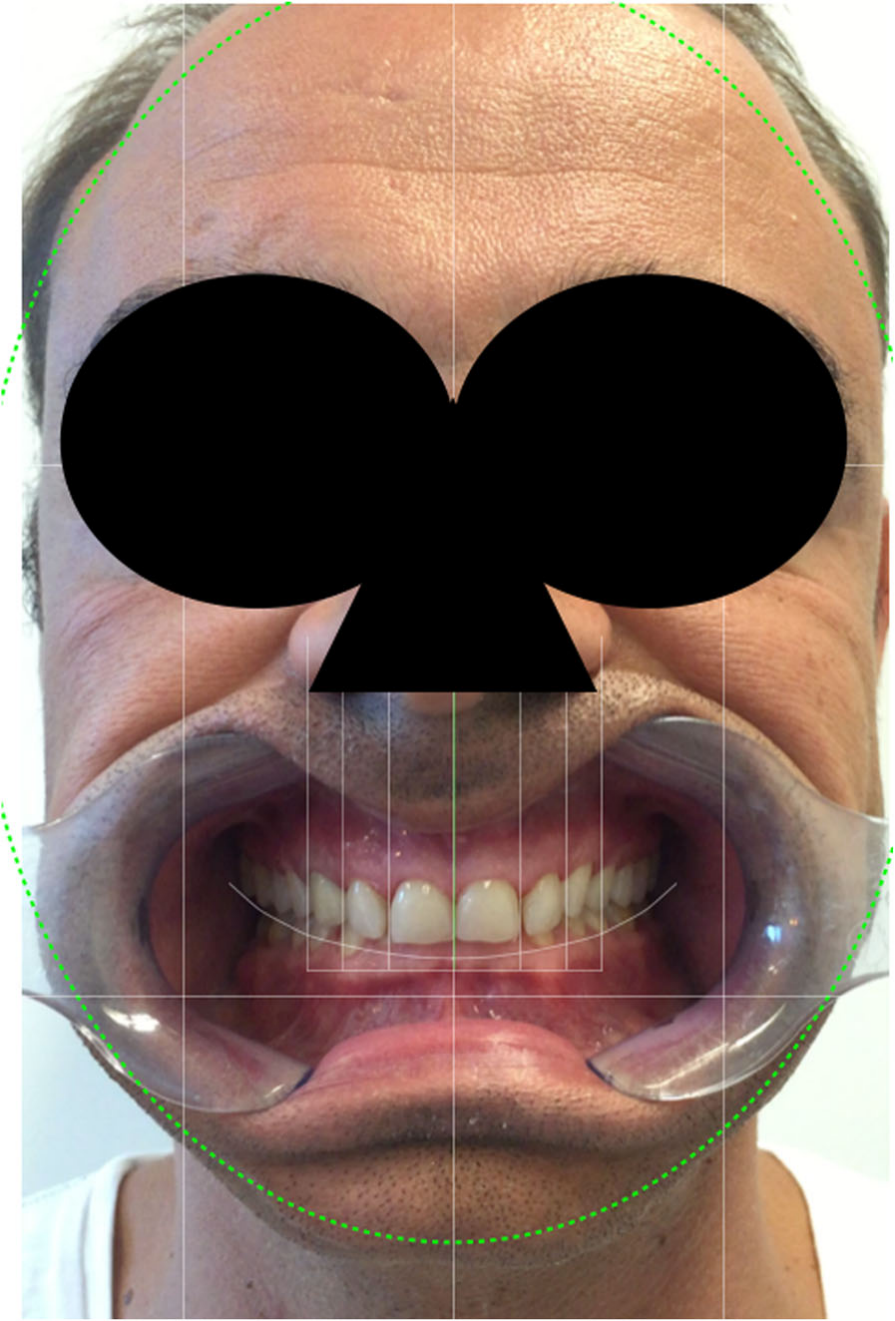
Digital Smile Design Protocol: smile picture with the retractors

**Fig. 5:**
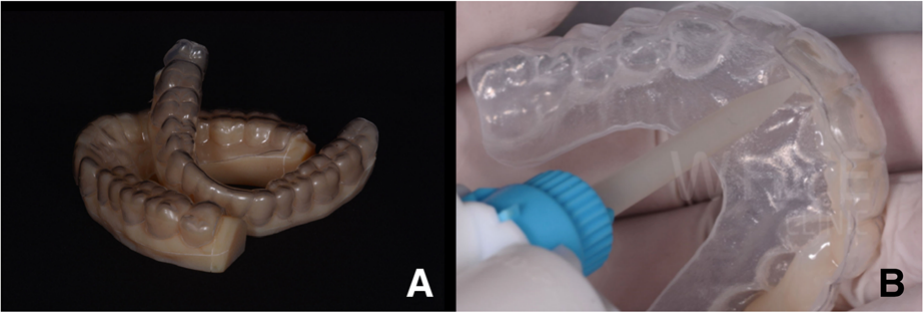
**a** Matrix for the motivational mock-up and digital models. **b** Motivational mock-up made with bisacryl (Structur VOCO, Germany)

**Fig. 6:**
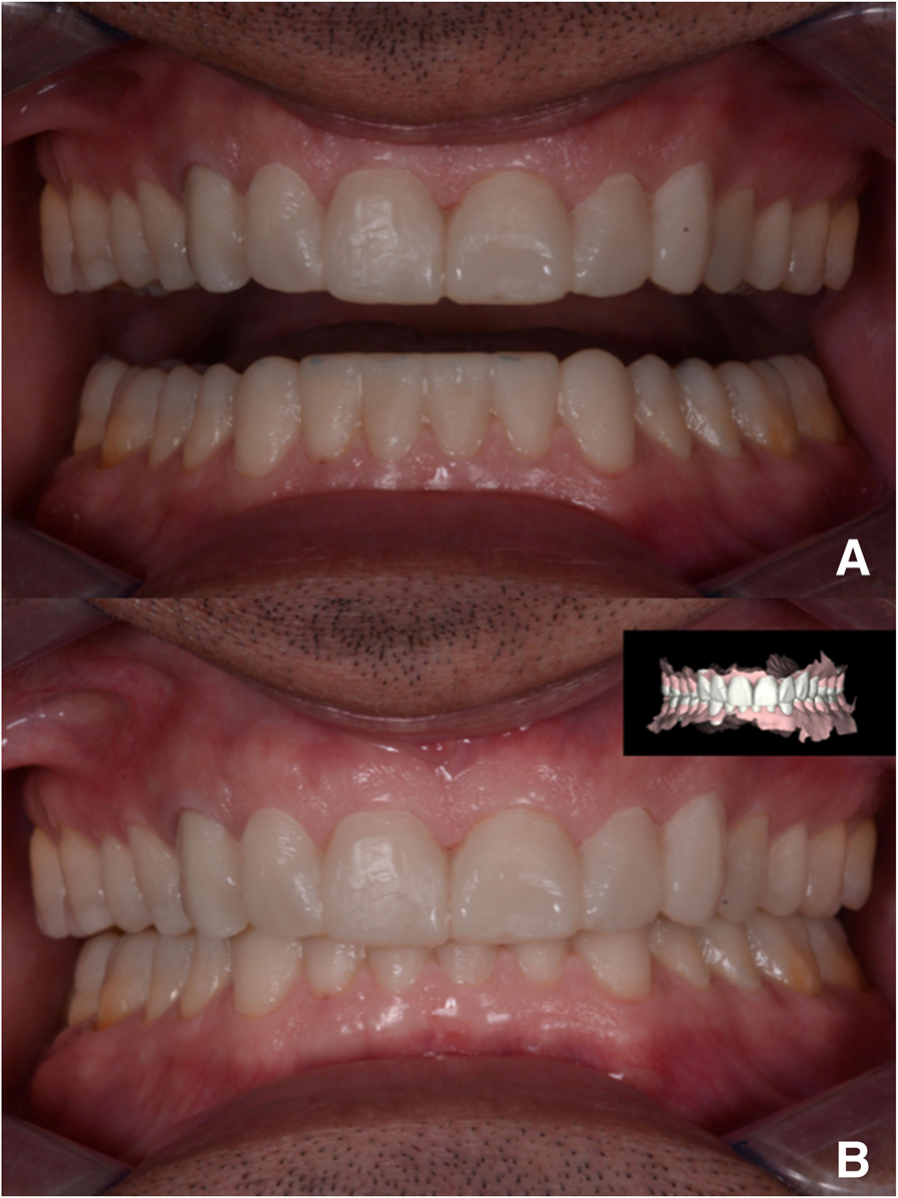
**a** Motivational Mock-up. **b** Motivational Mock-up and digital intraoral scan

Digital intraoral photographs were taken from a retracted frontal view, occlusal view and lateral view and extra oral photos (frontal, lateral and 45º) with a digital single lens reflex (DSLR) camera. A diagnostic impression of both arches was made with an intraoral scanner (Carestream 3500) as seen in Fig. 3. A maximum intercuspation position (MIP) was registered intra orally with the intraoral scanner (Carestream 3500), and the new vertical dimension of occlusion (VDO) was obtained by opening the appropriate amount on the virtual articulator in the CAD/CAM (computer-aided design/computer-aided manufacturing) software. The digital smile design (DSD) dynamic documentation protocol was applied: four videos were taken with a smart phone (iphone 6) from various calculated angles to achieve an ideal development of the facially smile frame. A facial frontal video with and without retractor smiling, a profile video, a 12 o’clock video and an anterior occlusal video perpendicular to the occlusal plan without mirror were recorded. Four more complementary videos, a facial interview, a 180º phonetics video, an intraoral functional and structural videos using a retractor, as seen in Fig. 4, were taken for functional, facial and structural analysis. The information was sent to the DSD Lab. The main goal of the DSD technique is to reconcile the photos of the three views (occlusal, frontal and 12 o’clock) with a digital ruler to create a smile frame supported by video analysis. Then a facially guided smile frame was created following these steps: digital facebow, smile curveshape and position, width determination using the recurring aesthetic dental (RED) proportion, length proportion, gingival curve, papillae curve, vermilion curve and arch curve. The 2D smile frame was turned into a 3D digital wax-up on CAD software. The final 3D file STL format was exported to a printer which generated the model with the new design. It was then used to fabricate a matrix for the motivational mock-up, made with bisacryl (Structur; VOCO), as seen in Fig. 5 and Fig. 6. In the new model, vertical dimension was augmented, so the patient spent two weeks with the provisional mock up to test the adaptation to the new vertical dimension (VD). After this bite was test driven, and since the patient was comfortable and stable, there was no need for further deprogramming the bite and defining a new centric relation (CR). With this new VD, the patient felt more comfortable and had no pain on the TMJ. The treatment plan was presented but, due to economic reasons, the patient did not want to continue the treatment.

**Fig. 7:**
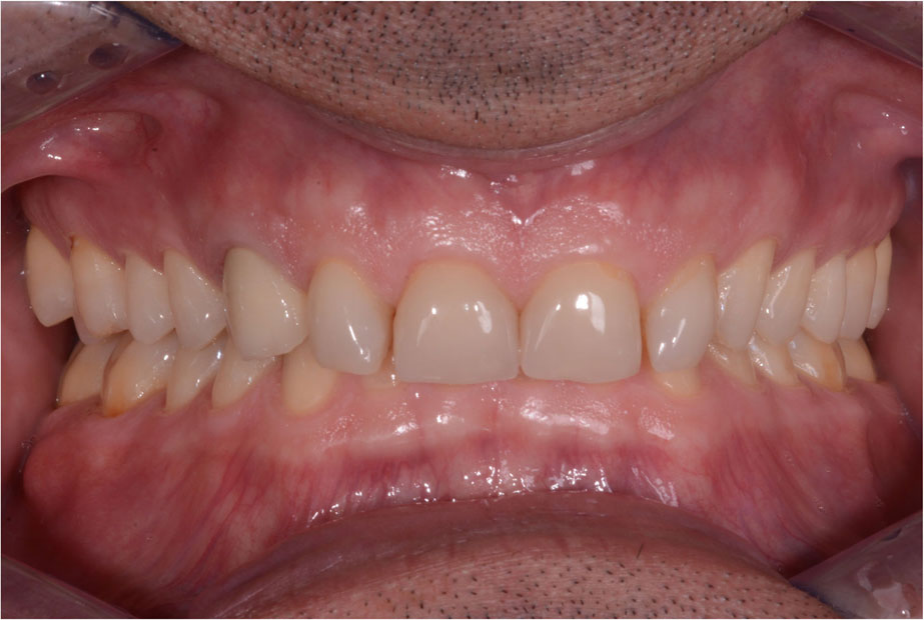
Two years later before treatment (2017)

**Fig. 8:**
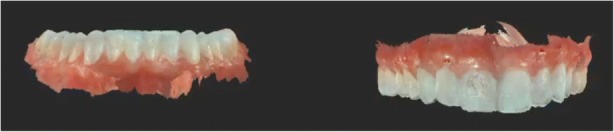
Pretreatment intraoral scan with Carestream® 3600 Rochester, NY, USA

**Fig. 9:**
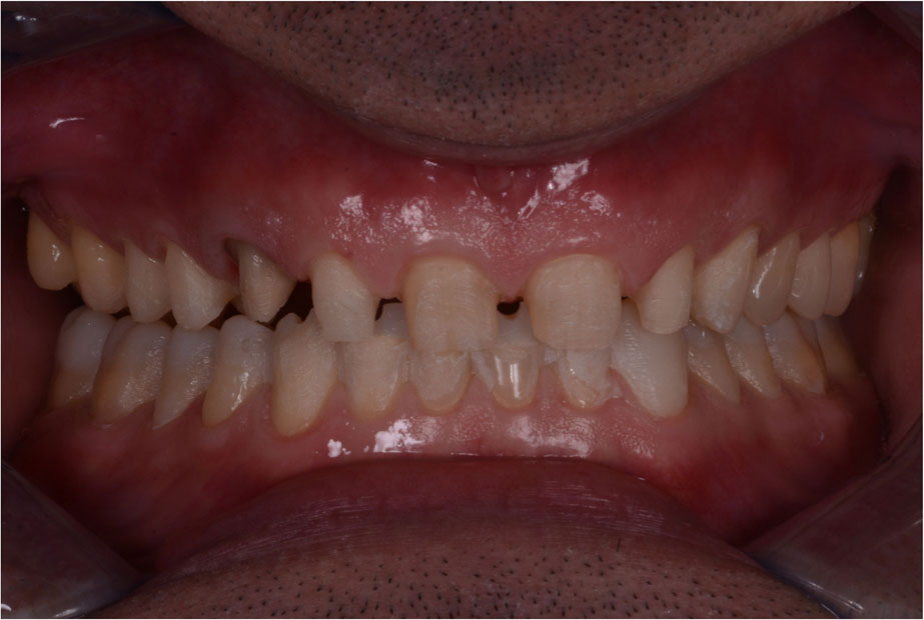
No preparation on the posterior teeth, minimal preparation on the anterior lower teeth, with the exception of 11, 12, 13, 21, 22, 23, that were already prepared

**Fig. 10:**
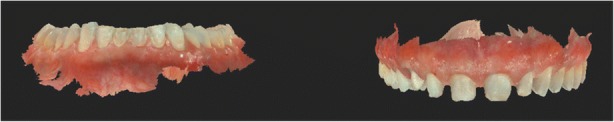
Digital scan of the abutment teeth preparation with Carestream® 3600; Rochester, NY, USA

**Fig. 11:**
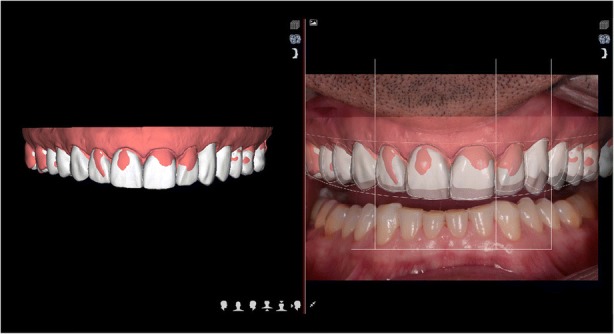
DSD planning

**Fig. 12:**
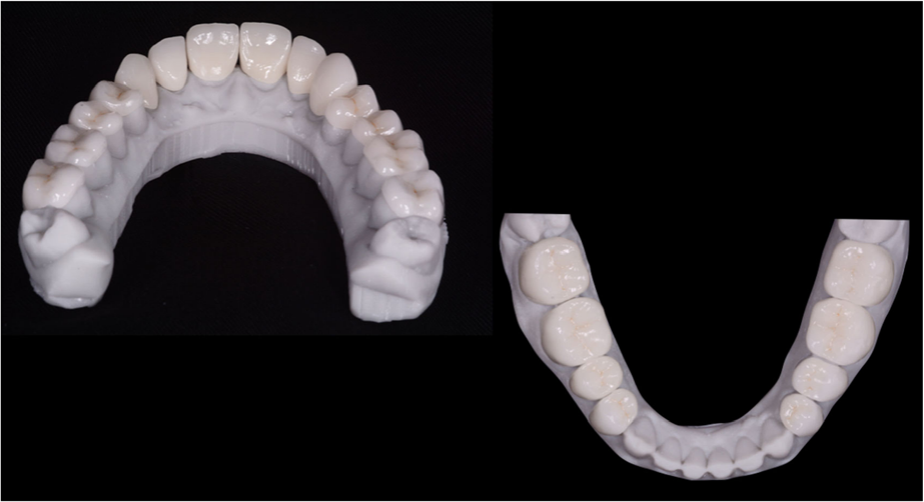
Definitive veneers and crowns prepared digitally using prosthetic software (Ceramill® mind) and fabricated in a milling machine (Ceramill® Motion 2) with machinable lithium disilicate ceramic blocks (VITABLOCS® TriLuxe forte for Ceramill® Motion 2)

In 2017 the patient returned to restart the treatment, as seen in Fig. 7, and a new intra oral scan (Carestream 3600) was made, as seen in Fig. 8. A new mock up for tooth preparation was made with bisacryl (Structur; VOCO) using a vaccum formed matrix (V-print Ortho clear; VOCO) printed by a 3D printer (Soflex; VOCO). Guided by the mock up, the abutment teeth were minimally prepared, as seen in Fig. 9. The old preparations from the second sextant teeth were maintained, no preparation in the posterior upper (14–17, 24–27) and lower teeth (34, 35, 36, 37, 44, 45, 46, 47), and a minimal preparation on the anterior lower teeth (31–33, 41–43) was made. A new intra oral scan (Carestream 3600) was made, as seen in Fig. 10. The information was sent to the DSD lab, as seen in Fig. 11, which then produced a STL file with virtual models that were sent and fabricated in the lab (Anatomic Lab). These 3D models (V-Print model; VOCO) were printed in a 3D printing machine (Solflex 650; VOCO). The definitive veneers and crowns were prepared digitally, using prosthetic software (Ceramill mind, Amann Girrbach), and fabricated in a milling machine (Ceramill Motion 2, Amann Girrbach) with machinable lithium disilicate ceramic blocks (VITABLOCS TriLuxe forte for Ceramill Motion 2, Amann Girrbach), as seen in Fig. 12.

**Fig. 13:**
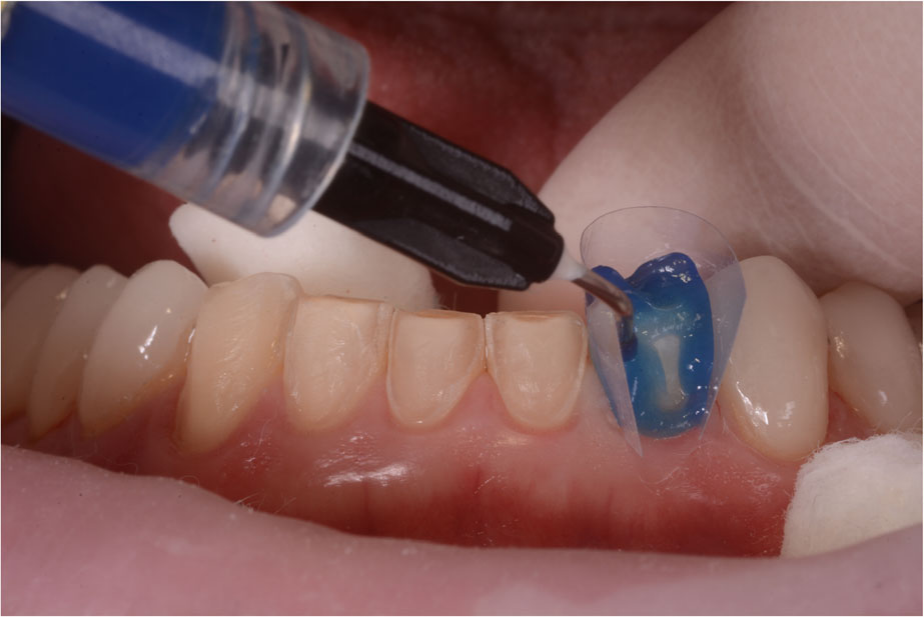
The crowns (11, 12, 13, 21, 22, 23) and veneers (14, 15, 16, 17, 24, 25, 26, 27, 31, 32, 33, 34, 35, 36, 37, 41, 42, 43, 44, 45, 46, 47) were adhesively luted to the abutments with a light polymerizing resin luting agent (Futurbond U and Bifix QM, VOCO, Germany)

**Fig. 14:**
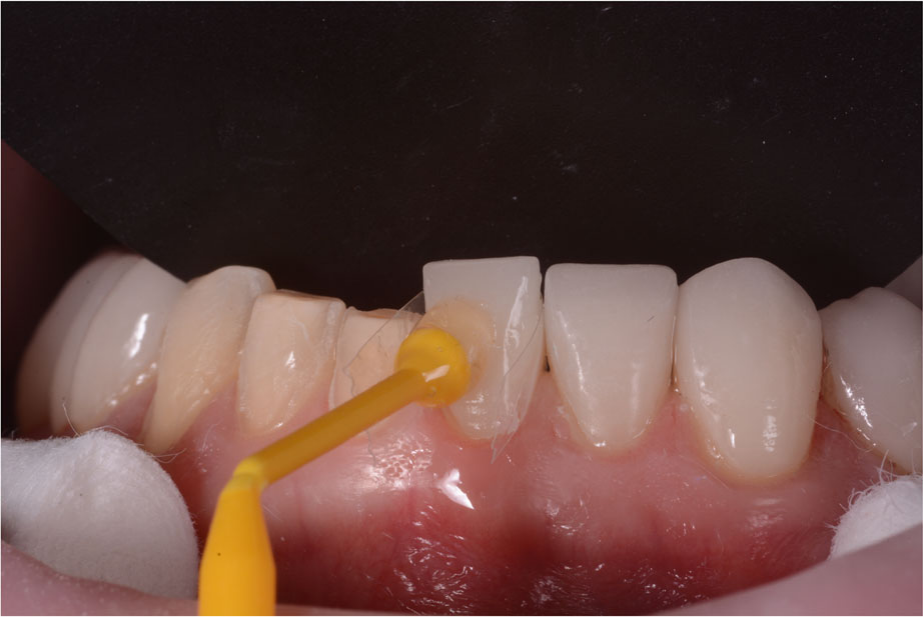
The crowns (11, 12, 13, 21, 22, 23) and veneers (14, 15, 16, 17, 24, 25, 26, 27, 31, 32, 33, 34, 35, 36, 37, 41, 42, 43, 44, 45, 46, 47) were adhesively luted to the abutments with a light polymerizing resin luting agent (Futurbond U and Bifix QM, VOCO, Germany)

**Fig. 15:**
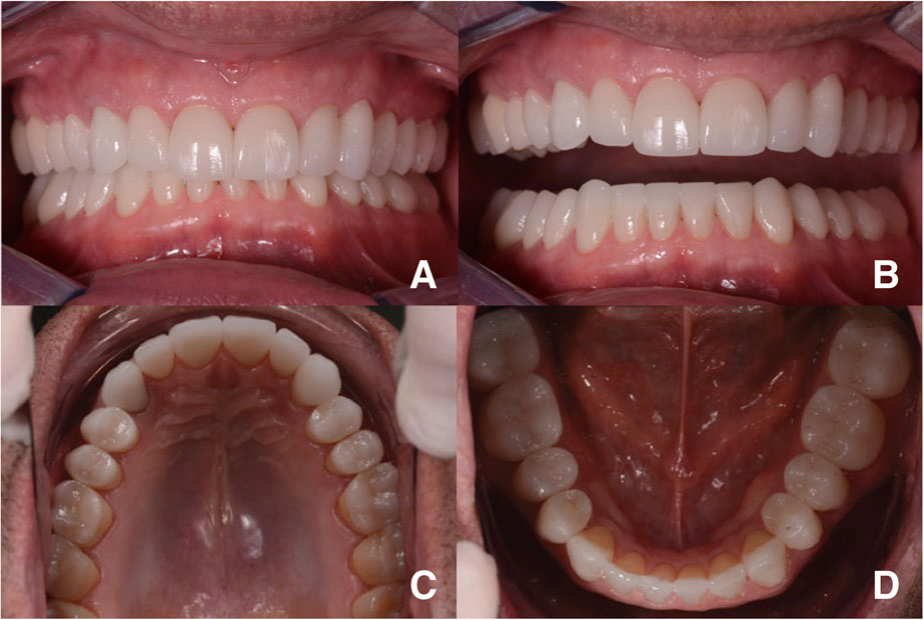
**a** and **b** Post-treatment intraoral photo. Front view. **c** Post-treatment intraoral photo. Occlusal view upper jaw. **d** Post-treatment intraoral photo. Occlusal view lower jaw

**Fig. 16:**
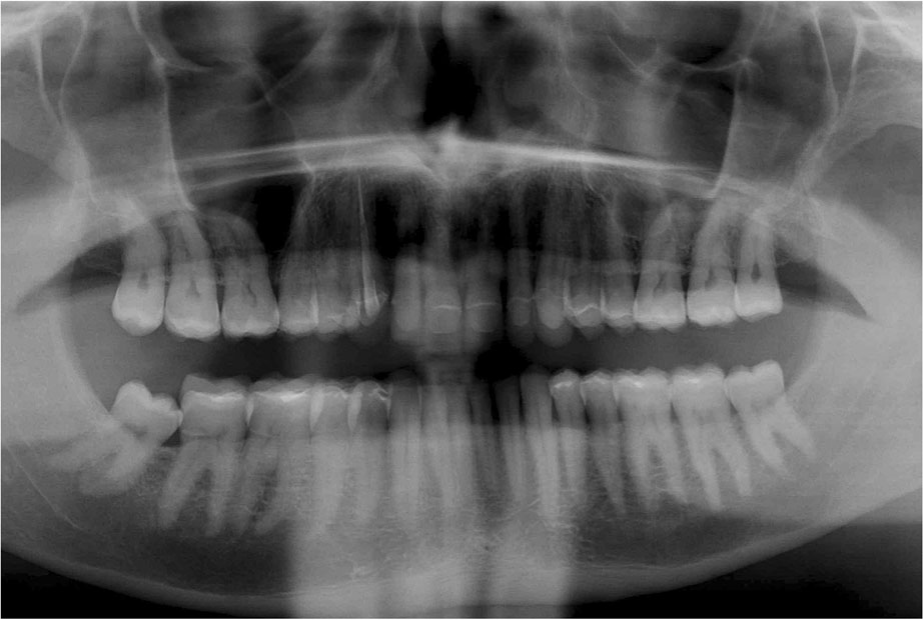
Final Panoramic

**Fig. 17:**
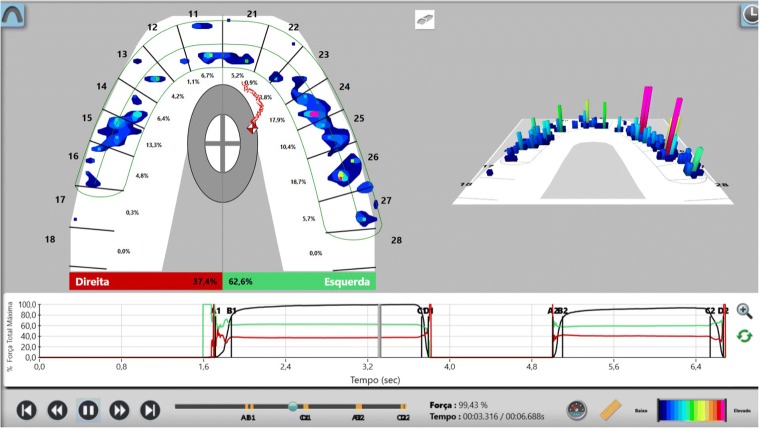
Occlusion confirmed using T-scan technology

After confirming marginal fit and optical properties in a trial insertion, isolation with a lip retractor (OptraGate, Ivoclar Vivadent) was applied. Following the manufacturer’s recommendations, the abutment teeth and ceramic crowns and veneers were prepared: the ceramic surface was prepared with aluminium oxide 50 μm, hydrofluoric acid 5% 20 s and rinsed 20 s, phosphoric acid 37% (Total etch, Ivoclar Vivadent) and alcohol 96% for cleaning, and silane 20 s (Monobond plus, Ivoclar Vivadent). The crowns (11–13, 21–23) and veneers (14, 15, 16, 17, 24, 25, 26, 27, 31, 32, 33, 34, 35, 36, 37, 41, 42, 43, 44, 45, 46, 47) were adhesively luted to the abutments using a light polymerizing resin luting agent (Futurbond U and Bifix QM; VOCO) polimerized by a high-power LED curing light device (Celalux 3; VOCO), as seen in Fig. 13 and Fig. 14. All the excess of the luting agent was removed and occlusal adjustments were done and confirmed using T-scan technology (T-scan; TeK-scan) as seen in Fig. 15a, b, c, and d, Fig. 16 and Fig. 17. A removable appliance in acrylic was made for protection of the final restorations.

After 6 months, final restorations were evaluated and they remained stable, without any fracture trait. Patient also referred that with the new vertical dimension he had no headaches.

## Discussion and conclusions

Due to the introduction of a whole range of devices, machines and software, the digital revolution is completely changing the dental profession. Thanks to the virtual world, we can plan in detail from surgical to restorative procedures, with the help of 3D modelling and software like CAD-CAM [14]. Intraoral scanners are digital devices used not only to obtain study models but also for the detection of impressions necessary for the modelling of a whole series of restorations [16]. Digital impressions are also a procedure that contribute to a more precise register of the bite, and they can eliminate several analogue procedures that can generate distortions [15].

In recent years, the variety of applications, together with the advantages of these machines, have made intraoral scanners highly interesting devices. Nowadays, there are several intraoral scanners in the market, but the most important element to be considered should be the accuracy, i.e. the quality of the data derived from scanning, a combination of trueness and precision [15, 16]. There are already reports in specialized literature that have studied the accuracy of the different intraoral scanners available [16–28]. In a recent in vitro study [20], the trueness and precision of four of the latest generation intraoral scanners have been compared. As a result of this study, the CS 3600 gave the best trueness results and therefore, this scanner was used to document our case report.

Facial analysis established solely on photographic evidence is incomplete and/or incorrect. Tarantili et al. also studied the smile on video and observed that the average duration of a spontaneous smile was 500 ms, which reinforces the difficulty of recording this moment in photographs [14]. Time can be saved by establishing a photo protocol. The documentation taken from photographs and videos allows for the creation of a 2D smile frame which is completely integrated into the face. The use of dynamic smile documentation, associated with the DSD protocol, will provide more efficient diagnoses, more consistent treatment plans and improved final results [14]. Thanks to a facially guided smile design using 3D software, and a planning center specialized in smile design, more dentists are able to deliver facially integrated rehabilitation.

Another advantage of a digital case is that generating a pre-op mock up allows the patient to see the impact of the new smile before committing to the treatment and irreversible procedures and increases patient education and case acceptance. Virtual treatment simulation also allows for the simulation of interdisciplinary procedures before starting the real treatment. This helps the clinician have better visualization of problems, a better decision-making process and less mistakes in the mouth.

Regarding the fabrication of final restorations, Cerec CAD/CAM machines are currently used to manufacture ceramic restorations based on computer-assisted design and produce a restoration on a single dental appointment. These restorations, commonly made with ceramic material, are becoming increasingly popular worldwide [29]. Recently CAD-CAM systems, especially digital impression systems, are uniting dental offices and dental labs, resulting in enhanced communication and restorative processes. Also, more affordable aesthetic restorations are produced by using CAD/CAM and natural shapes libraries.

Adhesive all-ceramic partial coverage restorations are also recognized as being a reliable treatment option for the posterior region. In this context, one should keep in mind that the majority of clinical long-term studies are based on leucite-reinforced glass-ceramics, whereas today, considerably stronger ceramic materials based on lithium disilicate are available [30–32]. The use of monolithic lithium disilicate material for restorations, reduces restorative failures as it eliminates layering and interfaces, which is usually the weak link between materials [31]. According to this case report, we chose to rehabilitate this patient using monolithic lithium disilicate material for restorations, since this case demonstrates that this material successfully works in a patient with bruxism when rehabilitated with the right VD.

All-ceramic onlays offer a sensible treatment option, since they permit a defect-oriented preparation method and eliminate the need for a retentive preparation design, thus bypassing conventional invasive treatment methods [33, 34]. In addition to eliminating the abrasion- and biocorrosion-inducing causes, restoring the aesthetic and functional properties and reconstructing the biomechanical properties of the affected teeth, they are considered the main treatment objectives. Furthermore, any restorative measures should be aimed at preventing any further pathologic wear in the long run. In this case report, minimal preparation of the posterior teeth was done to augment the VD and re-establish the aesthetics, and transitional restorations were made to test drive function and aesthetics of the patient and also allow for a minimally invasive treatment.

When establishing the new VD, by means of an anterior jig, the bite is deprogrammed and the centric relationship position is registered as a reliable starting point to achieve a comfortable and healthy inter maxillary position. Since the centric relation (CR) is a range and not one specific position, the OVD (occlusal vertical dimension) can then be fine-tuned, for more or less opening, in the Digital Articulator inside the CAD/CAM software (Exocad) based on the restorative and functional convenience, as described in this case report. Opening the bite inside the CR range will usually create clearance for a more conservative and simple restorative approach. But the limitation of opening will usually be determined by the anterior upper/lower tooth relationship since reasonable overbite/overjet is also a goal. In this case report, the facially guided smile design project (NemoDSD 3D) was exported into a CAD/CAM software (Exocad) to check the function in the digital articulator. The increase of the vertical dimension was determinated considering the CR range and the overjet/overbite of the patient.

The material of choice to substitute lost natural enamel is silicate ceramic due to its favorable optical and mechanical properties. However, minimally invasive veneer preparation, provisionalization, and adhesive bonding, requires greater expertise on the part of the operator when compared to complete coverage crown preparation and conventional cementation [35, 36].

Lithium disilicate is a material with excellent aesthetics and high strength (500 MPa, biaxial flexural strength), that can be used in minimally invasive preparation and adhesive cementation of crowns with a layer thickness of 1 mm [37]. In this case report we have used VITABLOCS TriLuxe forte for Ceramill Motion 2, Amann Girrbach, which have the indication for veneers, for partial and full crowns for the posterior area. We also wanted to prove in this case report that when the right VDO in a patient with bruxism is achieved, there is a low risk of fracture considering the high loading forces.

As a result, according to literature, indirect restorations combined with a removable appliance can be a solution for tooth ware and loss of the vertical dimension [38].

To sum up, thanks to the technology that was use in this case, we could obtain a fast an accurate result. Nevertheless, this study has some limitations. Firstly, this is a case report with a short-term follow-up. More cases with long term follow-up (up to 10 years) are needed to prove the success of this technique. Secondly, the material that was chosen in this rehabilitation can be consider a limitation. There is lack of literature that supports the use of lithium disilicate in posterior areas, since it is not the first indication, especially in patients with bruxism. Finally, we can also consider a limitation combining all this technology, since is still a big investment for dental practice nowadays, and it also still requires a learning curve to obtain an optimum result.

Thanks to the evolution of technology in dentistry, it is possible to do a full digital case and solve problems such as loss of vertical dimension successfully. Nevertheless, more clinical studies are needed to obtain consistent results about the digital work flow compared to the conventional technique in cases where there is loss of vertical dimension, and with long-term follow-up to closely follow the final restorations.

## Abbreviations

CAD-CAM: Computer aided design-computer aided manufacturing
CR: Centric Relation
DSD: Digital Smile Design
MIP: Maximum intercuspation position
RED: Recurring Aesthetic Dental
TMJ: Temporomandibular Joint
VD: Vertical Dimension
VDO: Vertical Dimension of Occlusion
